# On the Explanation of Born-Rule Statistics in the de Broglie-Bohm Pilot-Wave Theory

**DOI:** 10.3390/e20060422

**Published:** 2018-05-31

**Authors:** Travis Norsen

**Affiliations:** Department of Physics, Smith College, Northampton, MA 01063, USA; tnorsen@smith.edu

**Keywords:** pilot-wave theory, Bohmian mechanics, Born rule statistics, measurement problem

## Abstract

The de Broglie-Bohm pilot-wave theory promises not only a realistic description of the microscopic world (in particular, a description in which observers and observation play no fundamental role) but also the ability to derive and explain aspects of the quantum formalism that are, instead, (awkwardly and problematically) postulated in orthodox versions of quantum theory. Chief among these are the various “measurement axioms” and in particular the Born rule expressing the probability distribution of measurement outcomes. Compared to other candidate non-orthodox quantum theories, the pilot-wave theory suffers from something of an embarrassment of riches in regard to explaining the Born rule statistics, in the sense that there exist, in the literature, not just one but two rather compelling proposed explanations. This paper is an attempt to critically review and clarify these two competing arguments. We summarize both arguments and also survey some objections that have been given against them. In the end, we suggest that there is somewhat less conflict between the two approaches than existing polemics might suggest, and that indeed elements from both arguments may be combined to provide a unified and fully-compelling explanation, from the postulated dynamical first principles, of the Born rule.

## 1. Introduction

In standard textbook formulations of quantum mechanics, microscopic systems are described by wave functions which, under normal circumstances, obey Schrödinger’s equation. However, there are also abnormal circumstances in which the normal rules cease to apply. When a measurement occurs, for example, the system’s wave function momentarily ceases to evolve in accordance with Schrödinger’s equation and instead “collapses” to one of the eigenstates of the operator corresponding to the type of measurement being performed. Simultaneously, the outcome of the measurement comes to be registered in some directly-observeable (and separately-posited) classical object which we can think of as the pointer on the measuring device.

Which particular outcome is realized (i.e., to which particular eigenstate the quantum system’s wave function collapses and to which particular position the pointer ends up pointing) is supposed to be irreducibly random, with probability given by the Born rule: the probability for a particular eigenstate is given by the absolute square of the coefficient of that eigenstate in the linear expansion of the pre-measurement wave function.

For example, consider a position measurement on a quantum system with wave function ψ(x). The apparatus is initially in its ready state (including, say, a pointer with position $Y=Y_{0}$, resting at the left end of its scale next to an illuminated green light indicating that the device is able to make a measurement). The position operator $\hat{x}$ satisfies

(1) $$\hat{x}\;\delta \left(x-X\right)\;=\;X\;\delta \left(x-X\right),$$

i.e., the eigenfunctions of the position operator are the delta functions δ(x−X) corresponding to particles which are definitely located at x=X. The initial wave function of the quantum system can be written as a linear combination of these eigenfunctions as follows:

(2) ψ(x)=∫ψ(X)δ(x−X)dX.

Then, according to the quantum measurement postulates, during the measurement, the wave function of the quantum system collapses to δ(x−X) for some particular value of *X*, with probability (density) $P\left(X\right)={\left|\psi \left(X\right)\right|}^{2}$—and, simultaneously, the apparatus pointer jumps from $Y=Y_{0}$ to Y=λX (where λ is some proportionality constant capturing the calibration of the device) with, say, the light changing from green to blue to indicate that the measurement has been successfully completed.

More generally, if a measurement of the property corresponding to operator $\hat{A}$ is performed on a system with quantum state

(3) $$\psi =\sum_{m}c_{m}\psi_{m}$$

where $\hat{A}\psi_{m}=a_{m}\psi_{m}$, the probability that the measurement outcome is $a_{m}$ (and that the system is left in quantum state $\psi_{m}$) is

(4) $$P\left(a_{m}\right)={\left|c_{m}\right|}^{2}.$$

This is the Born rule.

Followers of orthodox (and/or, here equivalently, Copenhagen) quantum theory have tended to read allegedly deep truths about nature (such as the failure of determinism and the ineliminable dynamical role of observation in creating the observed reality) off from this account. More sober critics have instead regarded the orthodox formulation as obviously not providing the final truth, with the cluster of related implausible aspects of the orthodox formulation (for example, its division of the world into separate quantum and classical realms, with different kinds of ontologies and different dynamical laws, but also ad hoc exceptions to the usual dynamics when the two realms interact) being described as the “measurement problem” [1,2]. Recognition of the measurement problem has motivated the search for theories which cure it—or better, avoid it from the outset—by providing a description of the world (in both its ontological and dynamical aspects) that is *uniform* (i.e., consistent, coherent) and in which, in particular, “measurement” can be understood as simply another physical interaction to be treated just like any other, and with the orthodox measurement postulates (including especially the Born rule) becoming *theorems* derived from the more basic dynamical postulates.

There is thus a high degree of overlap between the quest to find a believable “quantum theory without observers” [3,4] and the quest to *explain* (rather than simply postulate) the Born rule. Perhaps the simplest such alternative theory to understand is the spontaneous collapse theories, in which Schrödinger’s equation is replaced by a stochastic differential equation that incorporates occasional, random, partial collapses which occur at a certain fixed rate per particle [5]. Such theories (approximately) reproduce the Born-rule randomness by, in effect, modifying the Schrödinger equation to include it [6]. So it is relatively easy to understand how the Born rule statistics arise in this kind of theory. By contrast, in the Everettian (a.k.a. “many worlds”) formulation of quantum theory [7], the collapse posulates of orthodox QM are eliminated entirely, with the wave function (of the universe) obeying Schrödinger’s linear equation all of the time. This cleanly removes the schizophrenia that gave rise to the measurement problem in the context of ordinary QM, but makes it rather difficult to understand how the Born rule might be derived—or even what the probabilities the Born rule is *about* could possibly *mean* in the context of a (deterministic!) theory in which every “possible” outcome is guaranteed to actually occur. This situation has given rise to a lively debate [8,9,10,11,12,13,14,15,16]. In addition, there have been many other attempts to similarly derive and/or explain Born’s rule, in the context of other alternative approaches to quantum theory, as well [17,18,19,20,21,22,23,24].

Here we will focus on the de Broglie-Bohm pilot-wave formulation of quantum theory [25]—a so-called “hidden variable theory” in which the usual quantum mechanical wave function (obeying Schrödinger’s equation) is supplemented with actual particle positions (A note on terminology: The phrase “hidden variable theory” is used here simply to denote a theory in which, contra orthodox QM, the complete description of a quantum system is given by a wave function *plus some additional variables*. However, as applied to the pilot-wave theory, the word “hidden” is rather inappropriate and misleading—Bell called it a piece of historical silliness [26]—since, if anything, it is the wave function and not the particle positions that are “hidden”. According to the theory, the world we literally see around us—including tables, chairs, other humans, planets, etc.—is composed of these particles, with the wave function playing a rather mysterious, invisible, background role of choreographing the particles’ motion). In terms of the simple example from a few paragraphs back, the pilot-wave theory can be understood as extending the quantum world all the way up into the macroscopic classical realm in the sense that the entire universe (including both the “quantum sub-system” and the “measuring apparatus”) will now be (partially) described by a wave function Ψ(x,y). The theory also extends the classical realm down into the microscopic quantum realm in the sense that now not only the particles composing the apparatus, but also the “quantum sub-system” particle, will be assigned definite positions (*Y* and *X*, respectively).

The introduction of additional variables (in particular the definite position *X* of the “quantum sub-system”) has often been regarded as pointless (or worse) in so far as the pilot-wave theory, at the end of the day, makes the same statistical predictions as ordinary QM. This concern is however wrong-headed for two (overlapping) reasons. First, to whatever extent one accepts that orthodox QM suffers from a measurement problem, one should be willing to (and should expect to) pay some price for its solution. It is ridiculous, that is, to dismiss the positing of new things, simply on the grounds of wanting as few things as possible, without considering what problems the new things are posited to solve. For example, should Pauli’s postulation of the existence of the neutrino have been dismissed, simply on the grounds that its introduction would make particle theory more complicated? However—second—it is not even clear that, overall, the pilot-wave theory is more complicated than orthodox QM. It is true that, as mentioned above, it extends the realm to be described in terms of wave functions from the microscopic to the entire universe, and extends the realm to be described in terms of particles with definite positions from the macroscopic to the entire universe. However, in so far as this supplemented ontology allows one to *derive*—rather than awkwardly postulate—the Born rule (and other rules about measurements), a strong case can be made that the pilot-wave theory is actually, overall, not more complicated but rather *simpler* than the orthodox formulation.

In the rest of the paper we attempt to explain and clarify this derivation of the Born rule in the context of the pilot-wave theory. We focus on the two recent, prominent, and fully-developed programs, ignoring for the most part their historical roots; [27] gives a helpful overview of some of the territory we will cover, including more historical references. Note also that virtually all of the issues to be discussed have close parallels in the context of (classical) statistical mechanics. We again want to focus on the explanation of Born rule statistics in the pilot-wave theory and avoid getting into generalized questions about the nature of statistical explanation, the relative merits of competing approaches to formulating and understanding the second law of thermodynamics, etc. However, readers who already know something about such controversial questions in the foundations of statistical mechanics will recognize parallel controversies playing out here. Readers who want to learn more about the foundations of statistical mechanics might see, for example, [28].

In the following section we give a more technical overview of the pilot-wave theory. Section 3 then provides an overview of one of the extant approaches to deriving the Born rule—the dynamical relaxation program of Antony Valentini. Some objections to Valentini’s program are reviewed in Section 4. Then, in Section 5 and Section 6 we review and then discuss some objections to the other extant program for deriving the Born rule—the “typicality” approach of Dürr, Goldstein, and Zanghí. The points discussed in these sections are illustrated with the results of numerical simulations for a simple example system. Finally, in Section 7, we take stock of the situation and suggest that elements from both the dynamical relaxation and the typicality programs are needed for—and make possible—a very clear and satisfying explanation of the origin of the Born rule in the context of the pilot-wave theory.

## 2. Pilot-Wave Theory and the Born Rule

The de Broglie-Bohm pilot-wave theory is best thought of as a candidate *explanation* of the measurement formalism of ordinary quantum mechanics, much as the kinetic/atomic theory (coupled with ideas from statistical mechanics) can be understood as the explanation of phenomenological thermodynamics. We begin here by reviewing the basic dynamical and ontological postulates of the theory as well as the “quantum equilibrium hypothesis” (QEH), the use of which allows a straightforward derivation of the standard quantum phenomenology. This overview is intended to establish a foundational context for the sketches of the two leading proposed analyses of the QEH which will be presented in the following sections.

For an *N*-particle system of, for simplicity, spinless, non-relativistic particles, the pilot-wave theory posits a wave function $\Psi \left(q,t\right)=\Psi \left({\vec{x}}_{1},{\vec{x}}_{2},\ldots ,{\vec{x}}_{N},t\right)$ obeying the usual Schrödinger equation

(5) $$i\hbar \frac{\partial \Psi }{\partial t}=-\sum_{i=1}^{N}\frac{\hbar^{2}}{2m_{i}}{\vec{\nabla }}_{i}^{2}\Psi +V\left({\vec{x}}_{1},{\vec{x}}_{2},\ldots ,{\vec{x}}_{N},t\right)\Psi$$

as well as *N* (literal, point) particles with configuration $Q\left(t\right)=\{{\vec{X}}_{1}\left(t\right),{\vec{X}}_{2}\left(t\right),\ldots ,{\vec{X}}_{N}\left(t\right)\}$ evolving according to

(6) $$\frac{dQ}{dt}=v\left(q,t\right){|}_{q=Q(t)}.$$

The configuration-space velocity field *v* is given by $v\left(q,t\right)=\{{\vec{v}}_{1}\left(q,t\right),{\vec{v}}_{2}\left(q,t\right),\ldots ,{\vec{v}}_{N}\left(q,t\right)\}$ where

(7) v→i(q,t)=ℏmiIm∇→iΨΨ=j→i(q,t)ρ(q,t).

Here ${\vec{j}}_{i}=\frac{-i\hbar }{2m_{i}}\left(\Psi^{*}{\vec{\nabla }}_{i}\Psi -\Psi {\vec{\nabla }}_{i}\Psi^{*}\right)$ is the *i*th particle component of the usual quantum “probability current”

(8) $$j\left(q,t\right)=\left\{{\vec{j}}_{1},{\vec{j}}_{2},\ldots ,{\vec{j}}_{N}\right\}$$

and

(9) $$\rho ={\left|\Psi \right|}^{2}$$

is the usual quantum “probability density”. Note, though, that despite these traditional names ρ and *j* have not yet been invested with any probabilistic significance, and should instead be thought of simply as properties of the pilot-wave field Ψ. (It is perhaps helpful here to regard ρ and *j* as analogous, respectively, to the “field energy density” and “Poynting vector” in classical electromagnetism. However, of course the fact that Ψ, and hence ρ and *j*, are functions on the 3N-dimensional configuration space, rather than 3-dimensional physical space, strains the analogy to some extent).

It is a purely mathematical consequence of Equation (5) and the definitions (8) and (9) that ρ and *j* jointly obey the continuity equation

(10) $$\frac{\partial \rho }{\partial t}+\nabla \cdot j=0$$

where $\nabla =\{{\vec{\nabla }}_{1},{\vec{\nabla }}_{2},\ldots ,{\vec{\nabla }}_{N}\}$. In light of Equation (7), this can be re-written as

(11) $$\frac{\partial \rho }{\partial t}+\nabla \cdot \left(\rho v\right)=0.$$

On the other hand, if at some initial time (say, t=0), the particle configuration *Q* is *random* with distribution P(q,0), then the distribution will evolve, under Equation (7), according to

(12) $$\frac{\partial P}{\partial t}+\nabla \cdot \left(Pv\right)=0.$$

The wave function “intensity” ρ and the particle probability distribution *P* thus evolve the same way in time and will hence remain identical for all times if they are equal at any one time. As this important fact is sometimes expressed, the particular distribution $P_{B}=\rho ={\left|\Psi \right|}^{2}$ is “equivariant”.

One should think here of the more familiar case of the Liouville distribution (that is, the Lebesque measure restricted to the energy surface in phase space) being “invariant” in classical statistical mechanics: The Hamiltonian flow guarantees that if the phase space point is Liouville-distributed at t=0 it will remain Liouville-distributed for all times. So the Liouville distribution has a dynamically privileged, equilibrium status, that is usually thought to play an important role in, for example, justifying the consideration of micro-canonical ensembles.

In the case of the pilot-wave theory, the analogous—dynamically privileged, equilibrium— distribution is not *in*variant, i.e., is not constant in time: $P_{B}$ will evolve non-trivially in time whenever Ψ does. However, the time-evolutions of Ψ and $P_{B}$ “track” one another, i.e., $P_{B}$ retains a constant relationship to Ψ even as both evolve: $P_{B}\left(q,t\right)={\left|\Psi \left(q,t\right)\right|}^{2}$. Hence “equivariance.”

The subscript “B” on $P_{B}$ is meant to stand for “Born”. This is because the assumption—that the initial (t=0) configuration of a quantum system was random with distribution $P_{B}$, i.e., the so-called “quantum equilibrium hypothesis” (QEH)—allows a straightforward proof that the pilot-wave theory reproduces the phenomenological predictions of ordinary quantum theory, i.e., the Born rule statistics. To see this, consider a typical measurement scenario involving a setup with generic configuration q={x,y} which we decompose into two parts representing the system-to-be-measured (*x*) and the measuring device (*y*). The measuring device should include a “pointer” whose final configuration indicates the outcome of the measurement. Suppose it is the property of the system corresponding to Hermitian operator $\hat{A}$ that is to be measured. Then (in order to justify calling the interaction a “measurement”) it must be that an initial state

(13) $$\Psi \left(x,y,0\right)=\psi_{m}\left(x\right)\phi_{0}\left(y\right)$$

(where $\psi_{m}\left(x\right)$ is an eigenfunction of $\hat{A}$ with eigenvalue $a_{m}$ and $\phi_{0}\left(y\right)$ represents the “ready” state for the measuring apparatus) evolves, under the Schrödinger equation, into

(14) $$\Psi \left(x,y,T\right)=\psi_{m}\left(x\right)\phi_{m}\left(y\right)$$

where $\phi_{m}\left(y\right)$ is “narrowly peaked” around configurations in which the apparatus pointer is pointing to the value “$a_{m}$”. (By “narrowly peaked” here we mean that $\phi_{m}^{*}\left(y\right)\phi_{n}\left(y\right)\approx 0$ whenever m≠n. That is, the functions $\phi_{m}\left(y\right)$ for distinct values of *m* have approximately non-overlapping support.)

Now suppose that the initial state of the system-to-be-measured is instead an arbitrary superposition of eigenfunctions, i.e.,

(15) $$\Psi \left(x,y,0\right)=\left(\sum_{m}c_{m}\psi_{m}\left(x\right)\right)\phi_{0}\left(y\right).$$

(Note, we assume here that things are normalized in the standard ways so that, for example, $\sum_{m}{\left|c_{m}\right|}^{2}=1$). It then follows, from the linearity of Schrödinger’s equation, that the post-measurement wave function will be

(16) $$\Psi \left(x,y,T\right)=\sum_{m}c_{m}\psi_{m}\left(x\right)\phi_{m}\left(y\right).$$

The post-interaction system-apparatus wave function is an entangled superposition which picks out no one particular measurement outcome. In ordinary quantum mechanics, where there is supposed to be nothing but the wave function, Equation (16) thus tends to lead to apoplexy, evasion, or the hand-waving introduction of new “measurement axioms”. However, in the pilot-wave theory, the actual measurement outcome is to be found, not in the wave function, but in the actual post-interaction configuration, *Y*, of the particles composing the apparatus pointer.

In addition, it is easy to see that if we adopt the QEH and thus regard the configuration Q(0)={X(0),Y(0)} as random and $P_{B}$-distributed, then, because of the equivariance property discussed above, the post-interaction configuration Q(T) will have distribution $P\left(q,T\right)=P_{B}\left(q,T\right)={\left|\Psi \left(x,y,T\right)\right|}^{2}$. Because of the property mentioned just after Equation (14), the cross terms will vanish and the marginal distribution for the pointer configuration *Y* becomes

(17) $$P_{T}\left[Y=y\right]=\int P_{B}\left(q,T\right)dx=\sum_{m}|c_{m}{|}^{2}{\left|\phi_{m}\left(y\right)\right|}^{2}.$$

Or equivalently, calling “$Y_{m}$” the set of (macroscopically indistinguishable) configurations in the support of $\phi_{m}\left(y\right)$, we can express this as follows:

(18) $$P_{T}\left[Y=Y_{m}\right]={\left|c_{m}\right|}^{2}.$$

That is: the probability is $|c_{m}{|}^{2}$ that, at the end of the experiment, the pointer indicates that the measurement had outcome $a_{m}$. This is of course just exactly the Born rule, Equation (4).

Please note that it may—and probably should—seem that there is hardly any distinction at all between the quantum equilibrium hypothesis (QEH) that we put in, and the Born rule that we get out. In effect, we put in the Born rule (for particle positions) at t=0, and get out the Born rule (for particle positions and so, in particular, for the positions of the particles composing the apparatus pointer) at t=T. Of course, it is noteworthy that the Born rule for particle positions is preserved in time (“equivariance”) and it is noteworthy that the Born rule for particle positions is sufficient to guarantee the generalized Born rule for arbitrary measurements. Still, though, to the extent that we simply assert the QEH as an additional postulate of the pilot-wave theory, the claim that the theory *predicts* Born rule statistics has a somewhat embarrassingly circular feel to it. This is why a genuinely convincing *derivation* of Born-rule statistics (in the context of the pilot-wave theory) needs to go a little deeper, by providing some kind of *justification* for the QEH. Let us then turn to exploring the two extant candidate justifications of this type.

## 3. The Dynamical Relaxation Justification

Following up an idea that was suggested by Bohm already in the 1950s [25,29], Antony Valentini proved, in his 1992 Ph.D. thesis [30] and associated papers [31,32,33], a “sub-quantum H-theorem” purporting to establish that non-equilibrium probability distributions will undergo a kind of dynamical relaxation toward coarse-grained equilibrium. This can be understood as a justification of the QEH, the idea being that even if, say, back at the big bang, particle positions were *not* $P_{B}$-distributed, they would inevitably become $P_{B}$-distributed (at least in a good-enough-for-all-practical-purposes, FAPP, coarse-grained sense) during the subsequent dynamical evolution of the universe, thereby justifying the application of the QEH to the “initial” conditions relevant to contemporary experimental investigations.

Let us review Valentini’s proof (Actually we will rehearse just one of several related arguments that he presents). Define g(q) as the *ratio* of $\rho ={\left|\Psi \right|}^{2}$ and the particle probability distribution *P*:

(19) $$g\left(q,t\right)\equiv \frac{\rho (q,t)}{P(q,t)}$$

and define the sub-quantum entropy “*S*” (which is really a kind of relative entropy between ρ and *P*) as

(20) S≡−∫dqPgln(g)=−∫dqρln[ρ/P].

To begin with, we note that in “quantum equilibrium” ρ=P, so g=1 and hence S=0. Also, since xln(x/y)≥x−y for all *x* and *y*, we have that

(21) $$S\leq -\int dq\left(\rho -P\right)=0.$$

Thus quantum equilibrium is a maximum (sub-quantum) entropy condition.

It follows from the fact that *P* and ρ obey the same continuity equations—(11) and (12)—that their ratio *g* is constant along particle trajectories. That is:

(22) $$\frac{dg}{dt}=\frac{\partial g}{\partial t}+\frac{dQ}{dt}\cdot \nabla g=0.$$

It is then a closely related fact that the exact (sub-quantum) entropy remains constant in time:

(23) $$\frac{dS}{dt}=0.$$

What Valentini showed, however, (following a long history of parallel demonstrations in the case of classical mechanics) is that an appropriately defined *coarse-grained* (sub-quantum) entropy can be shown to relax toward equilibrium.

Let us thus divide the configuration space up into cells of finite volume δV and define coarse-grained versions of the probability measure *P* and wave function intensity ρ as follows:

(24) $$\bar{P}\left(q\right)=\frac{1}{\delta V}\int_{\delta V}dq^{\prime }\;P\left(q^{\prime }\right)$$

and

(25) $$\bar{\rho }\left(q\right)=\frac{1}{\delta V}\int_{\delta V}dq^{\prime }\;\rho \left(q^{\prime }\right)$$

where, in both cases, the integration is over the cell containing the point *q*. We may also define a coarse-grained version of *g* as follows:

(26) $$\bar{g}\left(q\right)=\frac{\bar{\rho }\left(q\right)}{\bar{P}\left(q\right)}.$$

In terms of these quantities we may then also define the coarse-grained (sub-quantum) entropy

(27) $$\bar{S}=-\int dq\;\bar{\rho }\;\ln\left(\bar{g}\right).$$

Let us then consider the change in $\bar{S}$ from some initial time (0) to some later time (*t*):

(28) $$\bar{S}\left(t\right)-\bar{S}\left(0\right)=-\int dq\;\bar{\rho }\left(t\right)\;\ln\left[\bar{g}\left(t\right)\right]+\int dq\;\bar{\rho }\left(0\right)\;\ln\left[\bar{g}\left(0\right)\right]$$

Now comes an important assumption on the initial conditions: we assume what Valentini describes as “no ‘micro-structure’ for the initial state”, which basically means that the functions ρ(0) and g(0) are “smooth” and are therefore equal to their coarse-grained counterparts. Thus

(29) $$\bar{S}\left(t\right)-\bar{S}\left(0\right)=-\int dq\;\bar{\rho }\left(t\right)\;\ln\left[\bar{g}\left(t\right)\right]+\int dq\;\rho \left(0\right)\;\ln\left[g\left(0\right)\right].$$

However, then, the constancy of the exact *S* implies

(30) $$\bar{S}\left(t\right)-\bar{S}\left(0\right)=-\int dq\;\bar{\rho }\left(t\right)\;\ln\left[\bar{g}\left(t\right)\right]+\int dq\;\rho \left(t\right)\;\ln\left[g\left(t\right)\right].$$

The constancy of $\bar{g}$ and hence $\ln[\bar{g}]$ over the cells implies that $\int dq\;\bar{\rho }\left(t\right)\;\ln\left[\bar{g}\left(t\right)\right]=\int dq\rho \left(t\right)\ln\left[\bar{g}\left(t\right)\right]$ so that

(31) $$\bar{S}\left(t\right)-\bar{S}\left(0\right)=\int dq\;\rho \left(t\right)\;\ln\left(\frac{g(t)}{\bar{g}\left(t\right)}\right)=\int dq\;P\left(t\right)\;g\left(t\right)\;\ln\left(\frac{g(t)}{\bar{g}\left(t\right)}\right).$$

Finally, it can be shown that $\int dq\;P\;\left(\bar{g}-g\right)=0$ so we may add zero on the right hand side of the previous equation, yielding

(32) $$\bar{S}\left(t\right)-\bar{S}\left(0\right)=\int dq\;P\left(t\right)\;\left[g\left(t\right)\ln\left(\frac{g(t)}{\bar{g}\left(t\right)}\right)+\bar{g}\left(t\right)-g\left(t\right)\right].$$

The term in square brackets is (again using xln(x/y)≥x−y) non-negative, so

(33) $$\Delta \bar{S}=\bar{S}\left(t\right)-\bar{S}\left(0\right)\geq 0.$$

That is, the coarse-grained (sub-quantum) entropy *S increases* from its initial value.

Let us illustrate the implied dynamical approach to equilibrium with a numerical simulation. We follow [34,35] in considering a particle of mass *m* moving in a two-dimensional square box potential: V(x,y)=0 if 0<x<L and 0<y<L, and V=∞ otherwise. The energy eigenstates can be labeled with positive integers m,n with the energy eigenvalues given by

(34) $$E_{m,n}=\frac{\hbar^{2}\pi^{2}\left(m^{2}+n^{2}\right)}{2mL^{2}}.$$

**Figure 1:**
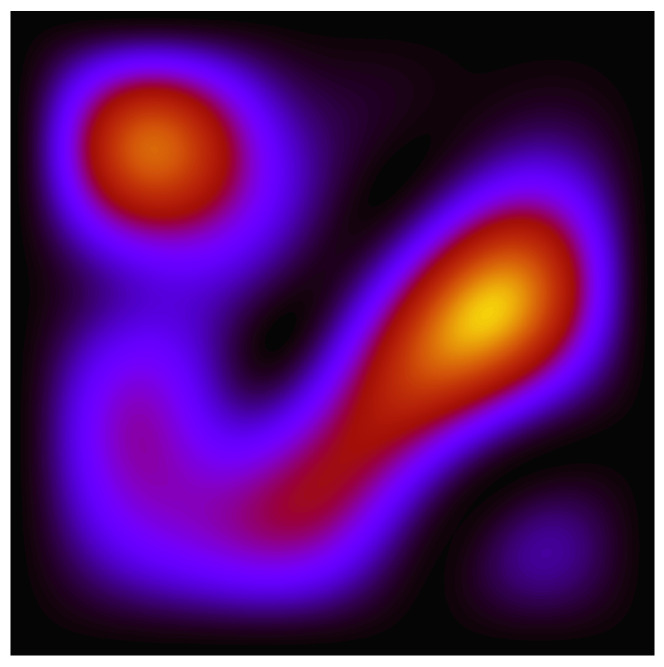
Density plot of $\rho \left(0\right)={\left|\Psi \left(0\right)\right|}^{2}$. Please note that ρ evolves periodically with period *T*. Note also that, for the particular non-equilibrium initial distribution P(0)=constant, g(0)=ρ(0)/P(0)=ρ(0). So this same figure can be taken also as illustrating g(0) for this particular non-equilibrium distribution. The corresponding g(t) for t=T, t=2T, and t=−T are shown in subsequent figures.

We take the initial quantum state to be a superposition (with equal amplitudes but randomly chosen phases) of the 6 lowest-lying energy eigenstates. The state Ψ(x,y,t) then evolves periodically with period $T=4mL^{2}/\hbar \pi$. At t=0 the wave function intensity $\rho \left(x,y\right)={\left|\Psi \left(x,y\right)\right|}^{2}$ is as shown in Figure 1.

As a concrete example of a non-equilibrium distribution, we will take the *uniform distribution* in which P(x,y) is constant (inside the “box”, and zero outside):

(35) $$P\left(x,y\right)=\left\{\begin{array}{cc} \frac{1}{L^{2}} & \mathrm{if}\;0<x<L\;\mathrm{and}\;0<y<L\\ 0 & \mathrm{otherwise} \end{array}\right.$$

Please note that the quantity g=ρ/P discussed above is simply proportional to ρ for P=constant, so Figure 1 may also be understood as a graph of g(x,y,0) for this particular non-equilibrium distribution.

**Figure 2:**
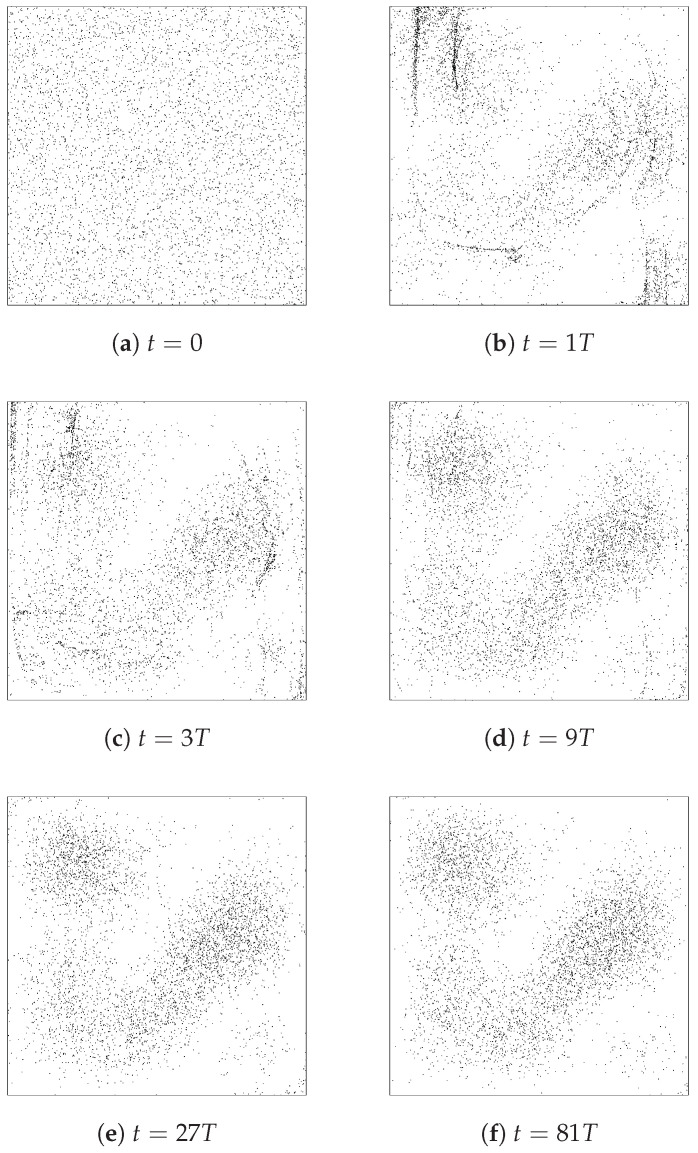
Relaxation of a uniform distribution toward coarse-grained equilibrium.

Valentini’s dynamical relaxation is perhaps best illustrated by considering the time-evolution of an ensemble of particles whose initial positions are chosen randomly in accordance with the non-equilibrium distribution *P*. The results of such a simulation, with an ensemble of 5000 particles, are shown in Figure 2. One can literally see that the initial t=0 distribution evolves steadily, over the course of several periods of the background wave function dictating the particle trajectories via Equation (6), from one that “looks uniform” to one that instead “looks ${\left|\Psi \right|}^{2}$”. Remember here that the wave function evolves in a periodic way, so ρ at each of the moments pictured is just the ρ shown in Figure 1.

In this simulation, these 5000 particles *appear* ${\left|\Psi \right|}^{2}$-distributed by (something of order) t=10T or maybe t=100T. However, the truth, of course, is that really the distribution is only approaching the P=ρ equilibrium in a coarse-grained sense; it is just that, for this particular system, the grain-size at which the dis-equilibrium can be seen happens to be smaller than the typical inter-particle spacing by t≈10T.

**Figure 3:**
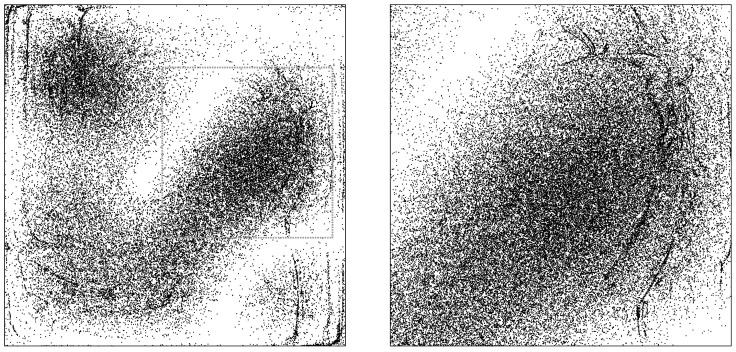
The left panel is the same as panel (d) of the previous figure, but for an ensemble of 50,000 initially-uniformly-distributed particles, allowing one to see some of the fine-grained structure in the distribution. The right panel zooms in on the portion of the box highlighted by the gray box on the left, for an ensemble of 200,000 particles, allowing even more fine-grained structure to be visible.

To illustrate this point, we can run another identical simulation, but with a much greater number or particles. Figure 3 thus shows, in the left panel, the same thing as Figure 2’s frame (d)—that is, an ensemble of initially-uniformly-distributed particles at t=9T—but with 50,000 instead of 5000 particles in the ensemble. The right panel shows a zoomed-in portion of the box for an even larger ensemble, making the fine-grained (dis-equilibrium) structure easily noticeable.

It is of course also possible to numerically track the evolution of P(x,y) through time. Compared to tracking an ensemble of particle positions, doing this in detail is somewhat computationally intensive. However, it gives a very clear sense of what is going on to see the evolution of an initially-constant *P* over just a few periods. Actually, it is somewhat more convenient to consider g=ρ/P since, as mentioned previously, the value of *g* is constant along particle trajectories. We can thus follow the numerical strategy introduced by Valentini and Westman to create a detailed map of P(x,y,t) as follows: consider a position X,Y where we want to know P(t); solve the equation of motion for the trajectory, backwards in time, to find the t=0 location $X_{0},Y_{0}$ of a particle which arrives at X,Y at time *t*; use the fact that *g* is constant along trajectories to assign

(36) $$g\left(X,Y,t\right)=g\left(X_{0},Y_{0},0\right)=\frac{\rho (X_{0},Y_{0},0)}{P(X_{0},Y_{0},0)}∼\rho \left(X_{0},Y_{0},0\right)$$

since, for the particular non-equilibrium distribution we consider, $P(X_{0},Y_{0},0)$ is a constant.

**Figure 4:**
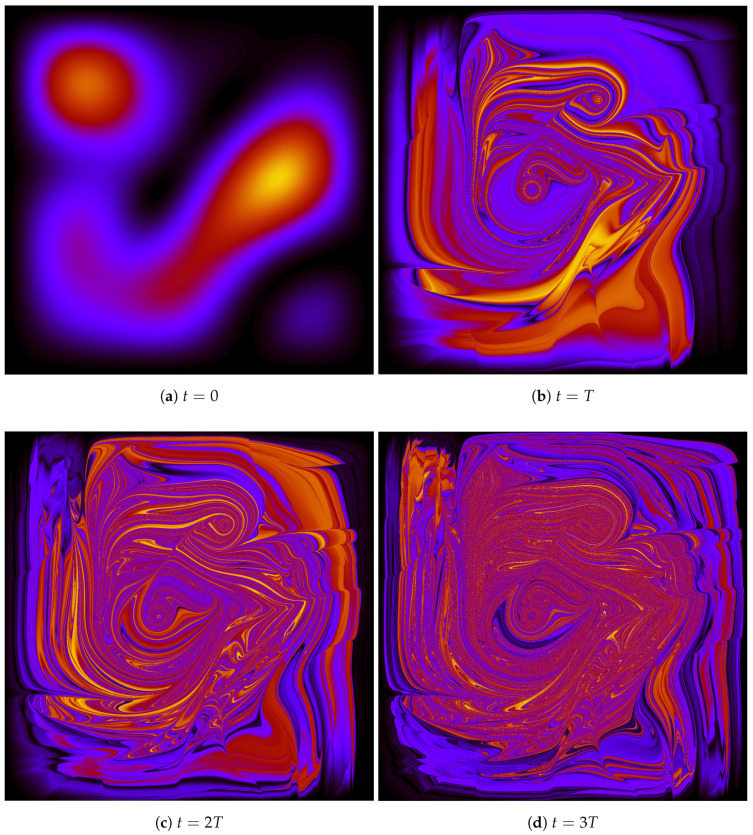
The time-evolution of g=ρ/P, for an initially-uniform *P*. The evolution through each period is like a “kneading” operation which results in the non-uniformities in *g* being systematically mixed down to smaller and smaller length scales. Further time-evolution would eventually result in a map whose $\bar{g}$ was uniform.

To really see the fine-grained-stucture in P(t), one already needs to consider an N×N grid of points X,Y, with *N* of order several thousand, after just a few periods (That is, already after just a few periods, the grain-size of the structure in *P* is of order $10^{-3}$ L). So computationally, making a beautifully detailed map of *g* is equivalent to running a simulation with something like 10 million particles. This is all by way of explaining why it would be computationally prohibitive to continue making detailed maps of *g* all the way out to, say, t=81T. However, as I remarked above, one nevertheless gets a very clear idea of what’s happening from looking at the evolution over just a few periods. This is illustrated in Figure 4.

Valentini characterizes the relaxation of $\bar{P}$ toward ρ—i.e., the relaxation of $\bar{g}$ to uniformity—as follows:

“The exact (fine-grained) density is given by [P=ρ/g]. Now, starting from an arbitrary [g(q,0)], the initial values of [*g*] are carried along the system trajectories in configuration space. If the system is sufficiently complicated, the chaotic wandering of the trajectories [Q(t)] will distribute the [*g*] values in an effectively random manner over the accessible region of configuration space. On a coarse-grained level, *P* will then be indistinguishable from [$\rho ={\left|\Psi \right|}^{2}$]. Another (equivalent) picture sees the increase of subquantum entropy as associated with the effectively random mixing of two ‘fluids’, with densities *P* and [ρ], each of which obeys the same continuity equation, and is ‘stirred’ by the same velocity field [so that] the two ‘fluid’ densities... will be thoroughly mixed, making them indistinguishable on a coarse-grained level”.

[31]

To summarize, the illustrations in this section clearly demonstrate that Valentini’s notion of dynamical relaxation to sub-quantum coarse-grained equilibrium *happens* in at least some systems. In particular we have seen how an initially *smooth g* (recall Valentini’s “no initial micro-structure” assumption) evolves toward a *g* with a very detailed, fine-grained structure which approaches perfect uniformity on a coarse-grained level. This is exactly in accordance with Valentini’s demonstration (and previous numerical simulations) and seems to add something quite relevant and substantial to our quest to understand the Born rule in the context of the pilot-wave theory.

In particular, as we discussed earlier, the invocation of the Quantum Equilibrium Hypothesis (QEH) at an initial time gives the explanation of Born rule statistics a somewhat circular appearance: you in effect put the Born rule in at t=0 only to get it back out again at a later time (corresponding, say, to the moment when some measurement process is completed). What Valentini’s sub-quantum H-theorem purports to show is that you do not need to postulate the QEH: a broader class of initial distributions, with P≠ρ, will relax towards P=ρ in a coarse-grained sense that is sufficient to account for the appearance of Born rule statistics in actual, finite-resolution experiments.

## 4. Objections to Dynamical Relaxation

If it could be shown that *any* possible non-equilibrium initial distribution P(q,0) would relax toward coarse-grained equilibrium for any possible initial wave function Ψ(q,0), that would of course constitute a rigorous derivation of the Born rule for the pilot-wave theory. However, it should be immediately clear that this is not possible.

For one thing, there certainly exist wave functions which generate particle trajectories that are insufficiently complicated/chaotic to produce relaxation—e.g., trivially, stationary states for which the velocity field is identically zero forever. For such a Ψ the distribution will stay constant, P(q,t)=P(q,0), and there can be no relaxation toward sub-quantum equilibrium. So at best the claim can be that we might expect relaxation toward coarse-grained equilibrium for the kinds of wave functions that might plausibly obtain in our world. There is, for example, some reason to think that the wave function of our universe is not a stationary state.

However, even for such appropriately non-trivial wave functions, it cannot be the case that *all* initial particle distributions relax monotonically toward equilibrium. This is clear from the time-reversal symmetry of the theory. We illustrate here with simulations paralleling those shown in the previous section, but now letting time run backwards from the initial t=0.

**Figure 5:**
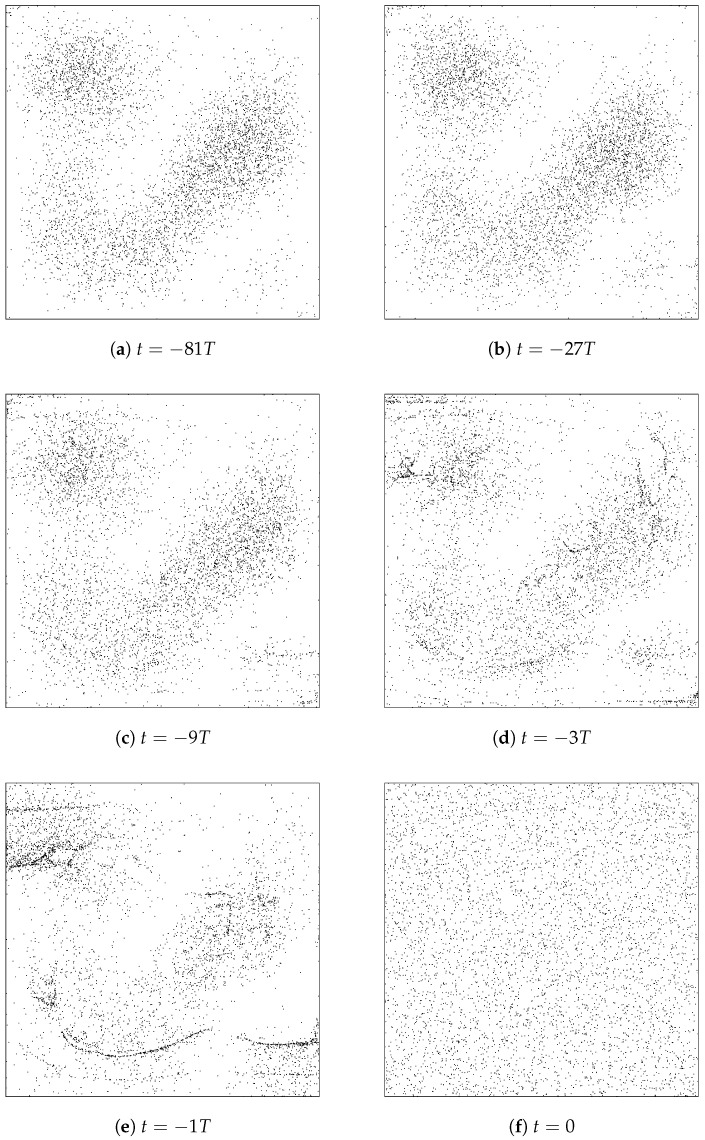
Anti-relaxation of an apparently-equilibrium distribution out of equilibrium!

Figure 5 shows the evolution of an ensemble of particles (with t=0 positions randomly chosen with probability distribution P(x,y,0)= constant) backwards in time. It is clear that the ensemble approaches coarse-grained equilibrium moving away from t=0 in the negative temporal direction, just as it does—recall Figure 1—in the positive temporal direction. Or, considering the evolution from negative times to t=0, as the images are arranged in the figure, one would say that an ensemble of particles that *appears* to have an equilibrium distribution (and is certainly in equilibrium in a coarse-grained sense) at one time may in fact evolve, at least for some considerable period of time, toward a dramatically non-equilibrium (here, uniform!) distribution.

**Figure 6:**
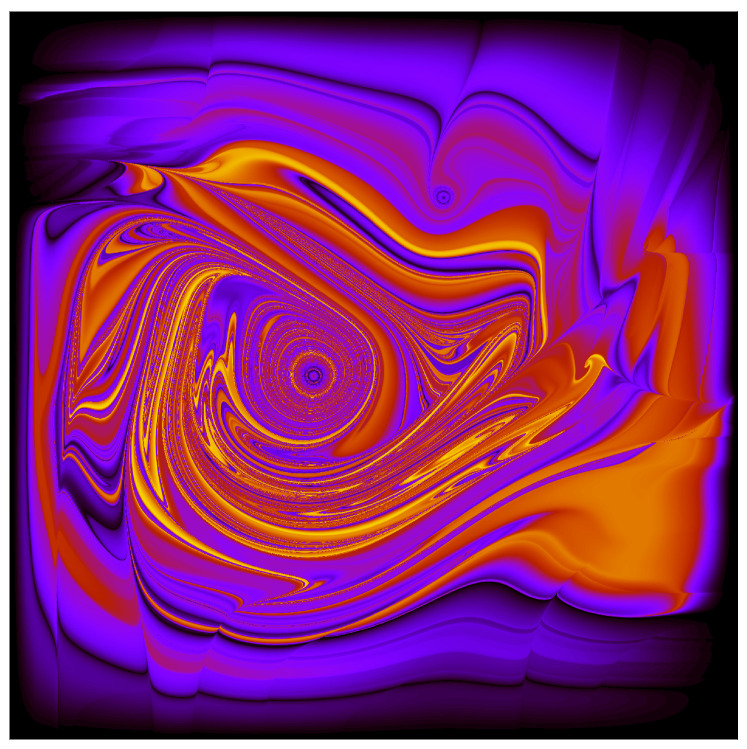
This is the distribution for g=ρ/P that is required at t=−T in order to make g=ρ, i.e., to make P= constant, at t=0. A very specific fine-grained micro-structure in *P* is required, at the earlier time, to generate a smooth, easily-describable non-equilibrium distribution at a later time.

This possibility is of course familiar from classical statistical mechanics as well (recall Loschmidt) and is not strictly speaking in conflict with Valentini’s sub-quantum H-theorem: the early-time ensemble depicted, for example, in frame (a) of Figure 5 is in fact not a typical ensemble associated with the equilibrium distribution P=ρ. Instead, it is a typical ensemble for a very different *P*—one which is equivalent to ρ in a coarse-grained sense, but which in fact contains incredible “detailed microstructure”. The flavor of this is conveyed by the image in Figure 6, which shows the distribution g=ρ/P, not at t=−81T but at t=−T, which forward-evolves into g=ρ (i.e., into P= constant) at t=0 (The distribution *g* that would be required at even earlier times is qualitatively similar, but of course with a considerably more fine-grained micro-structure). In any case, one can see that the initial distribution *P* for which the ensemble depicted in frame (a) of Figure 5 would be a typical example, will fail to respect Valentini’s “no initial fine-grained micro-structure” assumption, $P\left(q,0\right)=\bar{P}\left(q,0\right)$.

Callender has questioned whether this assumption of Valentini’s proof, that $P=\bar{P}$ initially, is any improvement over just assuming the quantum equilibrium hypothesis, namely, that P=ρ initially: “In both cases we are assuming that the early configuration distribution had a rather special profile” [27]. I think it would indeed be an improvement, at very least in the sense that it is a much weaker assumption: there are many possible “smooth” initial distributions satisfying $P=\bar{P}$ which do not correspond to coarse-grained equilibrium ($\bar{P}=\rho$).

Another set of possible concerns about the dynamical relaxation approach involves the distinction between continuous distributions *P*—whose relation, over time, to ρ is illustrated, for example, in Figure 4—and ensembles of particles with definite positions, as illustrated, for example, in Figure 2. It should be clear that if the quantum system we are studying is the universe as a whole—and the pilot-wave theory is fundamentally a theory about this ultimate system—then $\rho ={\left|\Psi \right|}^{2}$ (where Ψ is the universal wave function) is in some sense a really-existing thing, but *P*, the probability distribution over particle configurations *Q* of the universe as a whole, is not: what the pilot-wave theory posits as existing in addition to the universal wave function Ψ(q,t) is just some definite configuration $Q\left(t\right)=\left\{{\vec{X}}_{1}\left(t\right),{\vec{X}}_{2}\left(t\right),\ldots ,{\vec{X}}_{N}\left(t\right)\right\}$. There is no such additional thing as the probability distribution *P* over such configurations.

Callender criticizes Valentini on something like these grounds, namely for failing to clearly distinguish between the (effective) wave function ψ of an individual sub-system (within the universe) and the wave function Ψ of the universe. In particular, Callender stresses that “what is really needed is a justfication of” what, in our terminology, would be expressed as $p={\left|\psi \right|}^{2}$, i.e., a justfication for taking the probability distribution *p* associated with an ensemble of sub-systems with identical effective wave functions ψ to be ${\left|\psi \right|}^{2}$. Callender states that universal quantum equilibrium, $P={\left|\Psi \right|}^{2}$, is not sufficient for establishing what is really needed, namely, $p={\left|\psi \right|}^{2}$, and adds:

“Even if one proves that the universe as a whole is in quantum equilibrium, we really want to prove that patterns inherent in subsystems of the universe are in quantum equilibrium. Whether any [of Valentini’s results] survive the move to the proper understanding of [p=ρ] is not clear”.

[27]

I think Callender is on the right track about what is needed and is right to question whether Valentini’s argument provides it. That said, I do not think Callender quite puts his finger on the problem. If one could prove that “the universe as a whole is in quantum equilibrium”—$P={\left|\Psi \right|}^{2}$—I think it would indeed follow that ensembles of sub-systems with identical effective wave functions would also be in quantum equilibrium—$p={\left|\psi \right|}^{2}$. Indeed, Valentini demonstrates this very thing in Section 4 of [31].

The problem, though, is that it cannot be possible to prove that “the universe as a whole is in quantum equilibrium” because the “*P*” in “$P={\left|\Psi \right|}^{2}$” does not and cannot refer to anything real. That is, there is simply no such thing as the probability distribution *P* for particle configurations of the universe as a whole, because there is just one universe. This point, raised as a criticism of Valentini’s dynamical relaxation program, was made sharply by Dürr, Goldstein, and Zanghí: given the assumption that the configuration *Q* of the universe is random with probability distribution ${\left|\Psi \right|}^{2}$,

“you might well imagine that it follows that any variable of interest, e.g., *X*, has the ‘right’ distribution. However, even if this were so (and it is), it would be devoid of physical significance! As Einstein has emphasized, ‘Nature as a whole can only be viewed as an individual system, existing only once, and not as a collection of systems’.”

[36]

Coming fully to grips with this does not immediately render Valentini’s analysis irrelevant, for we can still attempt to understand the analysis as a description of ensembles of systems with identical effective wave functions. However, it does lead to several further points which I think are problematic for the relaxation approach to understanding Born rule statistics in the context of the pilot-wave theory.

Let’s start by straightening out the terminology a bit. Suppose the universe includes *n* sub-systems, with coordinates $x_{1},x_{2},\ldots ,x_{n}$, and that the universal wave function has the form

(37) $$\Psi =\psi \left(x_{1}\right)\psi \left(x_{2}\right)\cdots \psi \left(x_{n}\right)\Phi \left(y\right)+\Psi^{⊥}$$

where *y* denotes the coordinates of the environment of our collection of sub-systems, and $\Psi^{⊥}$ is macroscopically disjoint from Φ(y). In the pilot-wave theory we of course also have definite particle positions $X_{1}\left(t\right),X_{2}\left(t\right),\ldots ,X_{n}\left(t\right)$ and Y(t). If *Y* is in the support of Φ(y), then $\Psi^{⊥}$ will be irrelevant to the future motion of the particles and we may assign the effective wave function ψ to each of the *n* sub-systems.

We can define a (really-existing, meaningful) empirical distribution for the ensemble by taking

(38) $$p\left(x,t\right)=\frac{1}{n}\sum_{i=1}^{n}\delta \left(x-X_{i}\left(t\right)\right).$$

Valentini pursues this line in [30] (p. 18) and notes that “In the limit of large *n*, [p(x,t)] may for our purposes be replaced by a purely smooth function, again denoted just [p(x,t)], which in practical circumstances will behave like a probability distribution.” The relaxation analysis is then intended to show that the coarse-grained $\bar{p}$ approaches the Born-rule distribution $\rho ={\left|\psi \right|}^{2}$ for the sub-system.

Valentini acknowledges that

“[t]he very concept of a smooth distribution [*p*] is limited, being strictly valid *only in the purely theoretical limit of an infinite ensemble* (n→∞). This implies for example that in a laboratory consisting of a finite number of atoms, the actual distribution (say of electron positions) has the discrete form [of our (38), just above] so that one *necessarily* has some disequilibrium [$p\neq {\left|\psi \right|}^{2}$] on a fine-grained level”.

[30]

He acknowledges, that is, that for realistic finite ensembles the approach to equilibrium can in some sense only be approximate. This raises, for me, two concerns.

The first is that there would seem to be an additional, but unacknowledged, approximation that is relevant: the sub-system (effective) wave functions ψ will only evolve according to their own sub-system Schrödinger equations if the sub-systems are *perfectly* isolated from their environments. However, again, for more realistic ensembles of similarly-prepared systems, the isolation will be imperfect and hence the sub-system wave functions ψ for the individual ensemble members will not even evolve the same way in time. On the one hand, this renders certain key aspects of Valentini’s analysis as either questionable or downright inapplicable. For example, if, after some evolution time, the initially-identical effective wave functions for the individual ensemble members are no longer the same, there will not even exist any particular $\rho ={\left|\psi \right|}^{2}$ to which one might meaningfully compare the distribution $\bar{p}$! On the other hand, though, random variations in the evolution of the individual ensemble members’ effective wave functions (arising from random variations in the precise way in which each member is only imperfectly isolated from its environment) may constitute an additional source of noise that could accelerate the dynamical relaxation toward Born-rule distributions, in some appropriate sense. This issue deserves further study and attention.

However, there is a second and somewhat more fundamental worry associated with the fact that real ensembles will always be finite so that the distribution function *p* will be given by Equation (38) and hence not be a continuous function. The worry is not quite what Valentini acknowledges, namely that the fine-grained structure of the distribution *p* (for any realistic finite ensemble of identically-prepared sub-systems) implies “some disequilibrium ... on a fine-grained level”. Rather, the worry is that the fine-grained structure in *p* will mean that *p* cannot possibly equal its coarse-grained partner $\bar{p}$. However, that $p=\bar{p}$ at the initial time was one of the crucial assumptions of the theorem. So the worry is that the theorem is thus rendered simply inapplicable to realistic finite ensembles, i.e., irrelevant to goings-on in our universe.

In his 1991 paper, Valentini characterizes the “no fine-grained initial micro-structure” assumption as follows:

“It is this assumption which introduces a distinction between past and future: Essentially, it is assumed that there is no special ‘conspiracy’ in the initial conditions, which would lead to ‘unlikely’ entropy-decreasing behaviour”.

[31]

The basic problem that this leads us to seems to be the following: any *specific* initial condition for the configuration of the particles in our ensemble will possess “fine-grained initial micro-structure”, i.e., will be extremely ‘unlikely’—one might even say ‘conspiratorial’—just in the sense that, no matter which smooth probability distribution *p* one regards the realized configuration as having been drawn from, it will be one out of a continuously infinite set of possible such configurations, and hence highly “unlikely”.

The real question is: what *portion* of the continuously infinite set of possible configurations (each of them, considered alone, being extremely “unlikely”!) would lead to the troublesome “entropy-decreasing behaviour”? Do these “bad” initial configurations constitute rare exceptions to the typical behavior, or are the overwhelming majority of possible initial configurations of this “bad” sort, or what?

It is true, for example, that many possible initial configurations (for the ensemble of 2-D particle-in-a-box systems), drawn randomly from the probability distribution $p={\left|\psi \right|}^{2}$, will evolve into different configurations that still nevertheless have the property of being typical exemplars of $p={\left|\psi \right|}^{2}$, i.e., of looking like equilibrium ensembles. However, it is also true that there are initial configurations, drawn randomly from the probability distribution $p={\left|\psi \right|}^{2}$, which “lead to... entropy-decreasing behaviour”, i.e., which evolve into configurations that are extremely unusual, atypical exemplars of $p={\left|\psi \right|}^{2}$, i.e., which do not look like equilibrium distributions at all. Figure 5 of course provides a concrete example here.

Similarly, we have shown (by numerical simulation, see Figure 2) that many possible configurations, drawn randomly from a “non-equilibrium” distribution such as p= constant, evolve into configurations that appear to be in equilibrium, i.e., which have $\bar{p}={\left|\psi \right|}^{2}$. However, of course there will also exist initial configurations for the ensemble, that could be drawn randomly from p= constant, which do *not* evolve into something that looks equilibrated, but instead evolve into something that is even further from quantum equilibrium than p= constant.

All of these behaviours are possible and are realized for some of the continuously infinite set of possible configurations for the ensemble. To be genuinely convincing, then, an argument for “relaxation toward sub-quantum equilibrium” would need to establish that, in some appropriate sense, *most* of these possible non-equilibrium configurations will relax toward coarse-grained equilibrium. It would need to establish, in a word, that relaxation toward equilibrium is *typical*. I believe this is actually true: establishing it is very closely-related to what Valentini has already done, and anyway it is strongly reinforced by the numerical simulations we’ve used for illustrative purposes here and those more serious versions done by Valentini and Westman and Towler et al. in [34,35].

However, reframing what is needed from a relaxation argument in this way—in terms of typicality—also severely undercuts the *need* for a relaxation argument in the first place, in order to understand the origin of the Born rule statistics in the pilot-wave theory. Understanding that point is the subject of the following section.

## 5. The Argument from Typicality

J.S. Bell, in his 1981 essay “Quantum Mechanics for Cosmologists”, discusses the pilot-wave theory and the explanation of Born rule statistics therein. He notes that “it is easy to construct in the pilot-wave theory an ensemble of worlds which gives the [Born rule] exactly”. Given what we’ve called equivariance, “it suffices to specify... that the initial configuration [Q(0)] is chosen at random from an ensemble of configurations in which the distribution is” $P={\left|\Psi \left(q,0\right)\right|}^{2}$ [37].

Bell then continues, echoing some of the concerns raised in the previous section:

“However, this question arises: what is the good of ... giving distributions over a hypothetical ensemble (of worlds!) when we have only one world. The answer [... is that...] a single configuration of the world will show statistical distributions over its different parts. Suppose, for example, this world contains an actual ensemble of similar experimental set-ups. [...I]t follows from the theory that the ‘typical’ world will approximately realize quantum mechanical distributions over such approximately independent components. The role of the hypothetical ensemble is precisely to permit definition of the word ‘typical’.”

[37]

The idea, then, is that there is no need to try to explain how quantum equilibrium statistics could arise from some earlier out-of-equilibrium distribution; if the overwhelming majority of possible initial configurations of the universe will exhibit Born rule statistics—if Born-rule statistics are *typical*—then we should expect to see them and there is no further mystery to explain if we do.

We will give a brief rehearsal of the detailed argument, presented by Dürr, Goldstein, and Zanghí in 1992 [38], below. However, first, to help set the idea (which is often mis-understood), we discuss a couple of simple warm-up examples.

As a first example, suppose you pull a coin from your pocket and flip it 100 times. In addition, suppose that the particular sequence that you see happens to include roughly equal numbers of Heads and Tails (for example, 47 Heads and 53 Tails). How can you explain this fact? It would normally be regarded as a sufficient explanation to simply note that, of all the $2^{100}$ possible sequences you might conceivably have seen, *almost all* of them have “roughly equal numbers of Heads and Tails” (Some concrete numbers: there are approximately $10^{29}$ 100-flip sequences which have precisely 50 Heads, and about that same number again with 49, 48, and 47 Heads... whereas there is only a single sequence which has zero Heads. More than 99% of the $2^{100}$ possible sequences have between 35 and 65 Heads). The property of having “roughly equal numbers of Heads and Tails”, that is, is *typical* of 100-flip sequences. In addition, this means that, unless we have some specific relevant information about the way the flips were conducted (e.g., the coin is unevenly weighted and therefore highly biased towards Heads, or the “flips” were not really independent flips at all but were in some way highly correlated) we should not be surprised by the observed results. We saw, in short, just the kind of behavior that we should have expected to see, so there is nothing further to explain.

This same kind of reasoning is of course common and crucial in statistical mechanics. For example, consider the distribution of velocities for the *N* individual molecules in a certain macroscopic sample of (say, monatomic ideal) gas. Every student of thermodynamics learns that we should expect to observe the Maxwell velocity distribution

(39) $$P\left(\vec{v}\right)\;d^{3}v∼e^{-\alpha v^{2}}d^{3}v$$

(where $\alpha =m/2k_{B}T$), but what is the explanation for this? Again, the usual explanation is simply that the overwhelming majority of states that the gas might conceivably be in, will exhibit this distribution. Here is Boltzmann:

“... by far the largest number of possible velocity distributions have the characteristic properties of the Maxwell distribution, and compared to these there are only a relatively small number of possible distributions that deviate significantly from Maxwell’s”.

[39]

Of course, unlike the case of the sequence of coin flips in which the total number of sequences is finite, the number of distinct points in phase (or just velocity) space, consistent say with some constraint on the total energy of the gas, is continuously infinite. Any statement about properties exhibited by the “overwhelming majority” of the states thus presupposes a measure μ over the states.

The usual thing in the context of classical statistical mechanics is to take μ to be the restriction to the energy surface of the Lebesque measure on phase space; this measure is “natural” in the sense that it is invariant under the flow generated by the Hamiltonian equations of motion (Liouville’s theorem). It is then straightforward to prove that the overwhelming number of points on the energy surface exhibit the Maxwell velocity distribution. More formally, in the N→∞ limit, the μ-measure of the set of points for which the velocity distribution differs significantly from Equation (39), approaches zero. See [40] for a more detailed and very clear discussion.

Please note that it would be very strange, having shown that the Maxwell distribution is *typical* with respect to the measure μ, to worry that the specific choice of measure made any difference to the velocity distribution one regards as typical. Any other measure $\mu^{\prime }$ that is absolutely continuous with μ will, by definition, agree about the size of measure-0 and measure-1 sets. Of course, by hand-picking a measure that is concentrated on special points in phase space, one could diagnose any distribution one wants as “typical”. For example, consider the measure which is zero everywhere except at the point in velocity space where all *N* particles have the same velocity, ${\vec{v}}_{0}$:

(40) $$\mu^{\prime }=\delta^{3}\left({\vec{v}}_{1}-{\vec{v}}_{0}\right)\delta^{3}\left({\vec{v}}_{2}-{\vec{v}}_{0}\right)\cdots \delta \left({\vec{v}}_{N}-{\vec{v}}_{0}\right).$$

It would then follow that $\mu^{\prime }$-most of the accessible phase space points exhibit the highly non-Maxwellian velocity distribution

(41) $$P\left(\vec{v}\right)\;d^{3}v∼\delta \left(\vec{v}-{\vec{v}}_{0}\right)$$

in which all particles have identical velocities ${\vec{v}}_{0}$. However, such games are as transparently ridiculous as they are possible. The idea is that any “reasonable” measure—any measure which is not specifically hand-tailored to give special weight to phase space points not exhibiting the Maxwell distribution—will agree with the “natural” measure about the Maxwell distribution being typical.

In the case of the pilot-wave theory, in which the state of the universe at a given moment *t* is given by the universal wave function Ψ(q,t) and the particle configuration Q(t), the natural measure of typicality is the one given by $\mu ={\left|\Psi \right|}^{2}$. The equivariance property discussed above means that, although μ itself will be time-dependent (because Ψ is time-dependent), the form “$\mu ={\left|\Psi \right|}^{2}$” will be timelessly true: $\mu \left(q,0\right)={\left|\Psi \left(q,0\right)\right|}^{2}$ implies $\mu \left(q,t\right)={\left|\Psi \left(q,0\right)\right|}^{2}$ for all *t*. (The uniqueness of this equivariant measure was established in [41].)

We may divide the universe into a sub-system of interest (with degrees of freedom *x*) and its environment (with degrees of freedom *y*), so q=(x,y) and Q=(X,Y). It is then trivial to derive what Dürr, Goldstein, and Zanghí call the “fundamental conditional probability formula”

(42) $$\mu \left(X_{t}\in dx|Y_{t}\right)={\left|\psi \left(x,t\right)\right|}^{2}\;dx$$

where

(43) $$\psi \left(x,t\right)=\frac{\Psi (x,Y(t),t)}{{\int |\Psi \left(x,Y\left(t\right),t\right)|}^{2}\;dx}$$

is the (normalized) conditional wave function (CWF) for the sub-system.

It is reasonable to think of the CWF as the wave function of the sub-system, in the context of the pilot-wave theory, for at least two reasons: first, the guidance formula for the sub-system configuration *X* can be written directly in terms of ψ(x); and second, the CWF will obey the obvious sub-system Schrödinger equation when the sub-system is suitably decoupled from its environment. (Note, however, that in general the CWF does *not* obey a simple sub-system Schrödinger equation, but instead evolves in a more complicated way; this is a feature, not a bug, since the complicated non-linear evolution in fact reproduces, in a precise and continuous way, the complicated non-linear evolution that would be predicted, in the context of ordinary QM, by the *ad hoc* combination of Schrödinger evolution and intermittent applications of the collapse postulate). In the simple case that the full system wave function is a product,

(44) Ψ(x,y)=ψ(x)ϕ(y),

the CWF for the *x* sub-system coincides with ψ(x). One should thus appreciate that the CWF is a generalization of the ordinary quantum mechanical wave function: the Bohmian CWF agrees with the wave function that would be attributed to a system in ordinary QM *in those situations where ordinary QM would attribute any definite wave function at all to the sub-system*; but the Bohmian CWF always exists and provides a rigorous interpolation (consistent with the overall Bohmian dynamics for the particles) between such times, even through preparations and measurements. Note also that, in the situations where the *effective* wave function for a sub-system exists, it is given by the CWF, so we use those interchangeably here.

To understand Dürr, Goldstein, and Zanghí’s main statistical result, let us consider again the situation described in Section 3 in which the universal wave function has the structure given in Equation (37). Suppose that $\Psi^{⊥}$ and Φ have macroscopically disjoint *y*-supports and that $Y_{t}\in supp\left(\Phi \right)$ so that each of the *n* sub-systems has identical CWF ψ. It then follows from the fundamental conditional probability formula that

(45) $$\mu \left(X_{1}\in dx_{1},X_{2}\in dx_{2},\ldots ,X_{n}\in dx_{M}|Y\right)=|\psi \left(x_{1}\right){|}^{2}\cdots {\left|\psi \left(x_{n}\right)\right|}^{2}\;dx_{1}\cdots dx_{n}.$$

Thus, the configurations $X_{i}$ of the particles composing the members of our ensemble of identically-prepared sub-systems are independent, identically distributed random variables, with common distribution ${\left|\psi \left(x\right)\right|}^{2}$.

It is then essentially an immediate and standard application of the law of large numbers to infer that, in the n→∞ limit, the empirical distribution

(46) $$p_{emp}\left(x\right)=\frac{1}{n}\sum_{i=1}^{n}\delta \left(x-X_{i}\right)$$

is very close to $\rho ={\left|\psi \right|}^{2}$ for μ-most initial configurations Q(0) (A slightly more precise statement is that the measure of the “agreement set” for which $\left|\right|p_{emp}-\rho \left|\right|\leq \epsilon$, for a suitable notion of ||·||, approaches unity as n→∞. Note also that, in addition to the “equal time analysis” we have sketched here, Dürr, Goldstein, and Zanghí provide in addition an analysis of the statistics of measurements performed on sub-systems across time; they show that, in this case as well, Born-rule statistics are typical. See [38] for elaboration and details).

The argument is thus completely parallel to the standard statistical-mechanical explanation of the Maxwell velocity distribution sketched above: using an appropriate natural measure over the space of possible states, it is possible to infer that the overwhelming majority of those states will realize a certain statistical property—namely, that ensembles of sub-systems, each member of which is prepared with (effective) wave function ψ, should exhibit Born-rule statistics $p={\left|\psi \right|}^{2}$. One should therefore expect the positions of particles in identically-prepared quantum mechanical sub-systems to be Born-rule distributed, according to the pilot-wave theory, for exactly the same reason that one should expect, in the context of classical mechanics, to see a Maxwell velocity distribution: “... by far the largest number of possible ... distributions have [this characteristic] distribution, and compared to these there are only a relatively small number of possible distributions that deviate significantly from [it].”

There is thus no need, according to the proponents of the typicality program, to explain how “quantum equilibrium” might have arisen, via relaxation, from some earlier non-equilibrium distribution (and so whether, in particular, relaxation toward equilibrium is typical for non-equilibrium distributions). If “by far the largest number of possible ... distributions” exhibit Born rule statistics, then there is precisely the same motivation for expecting non-Born-rule distributions in, say, the early universe as there is for expecting them today—namely, none.

## 6. Objections to the Typicality Argument

Proponents of the typicality argument insist that the measure μ be thought of (merely) as a measure of typicality and not as a fully detailed probability distribution. They stress, that is, that in the argument the *only* use to which μ is put is in assessing certain sets as having μ-measure of approximately unity (or approximately zero) (See [42] for further discussion).

It must be admitted, though, that the distinction between regarding $\mu ={\left|\Psi \right|}^{2}$ as a *typicality* measure, and regarding it instead as a full-fledged *probability* measure, is rather subtle and perhaps has the air of a distinction without much of a difference (Valentini, for example, writes in a footnote of [43]: “Note that if the word ‘typicality’ is replaced by ‘probability’, the result of Dürr et al. becomes equivalent to the ‘nesting’ property proved by Valentini, which states that an equilibrium probability for a many-body system implies equilibrium probabilities for extracted subsystems...”) (Indeed, such skepticism about the importance, or meaningfulness, of the difference is perhaps supported by the name Dürr, Goldstein, and Zanghí give to Equation (42)). The most common and important objection to the claim that Born-rule statistics are *typical* (and hence not in need of some special explanation involving, for example, dynamical relaxation toward equilibrium) is thus that the argument purporting to establish this is *circular*: we only get Born-rule statistics out for subsystems, according to this objection, because we assume Born-rule statistics apply to the universe as a whole.

In [43], for example, Valentini writes that the approach of Dürr et al.

“may be illustrated by the case of a universe consisting of an ensemble of *n* independent subsystems (which could be complicated many-body systems, or perhaps just single particles), each with wavefunction $\psi_{0}\left(x\right)$. Writing $\Psi_{0}^{univ}=\psi_{0}\left(x_{1}\right)\psi_{0}\left(x_{2}\right)\cdots \psi_{0}\left(x_{n}\right)$ and $X_{0}^{univ}=\left(x_{1},x_{2},x_{3},\ldots ,x_{n}\right)$, a choice of $X_{0}^{univ}$ determines – for large *n* – a distribution $\rho_{0}\left(x\right)$ which may or may not equal $|\psi_{0}{\left(x\right)|}^{2}$ .

“Now it is true that, with respect to the measure $|\Psi_{0}^{univ}{|}^{2}$, as n→∞ almost all configurations $X_{0}^{univ}$ yield equilibrium $\rho_{0}={\left|\psi_{0}\right|}^{2}$ for the subsystems. It might then be argued that, as n→∞, *dis*equilibrium configurations occupy a vanishingly small volume of configuration space and are therefore intrinsically unlikely. However, for the above case, with respect to the measure $|\Psi_{0}^{univ}{|}^{4}$ almost all configurations $X_{0}^{univ}$ correspond to the disequilibrium distribution $\rho_{0}={\left|\psi_{0}\right|}^{4}$. This has led to charges of circularity: that an equilibrium probability density $|\Psi_{0}^{univ}{|}^{2}$ is in effect being assumed for $X_{0}^{univ}$; that the approach amounts to inserting quantum noise into the initial conditions themselves...”

In [44], Jean Bricmont rehearses a closely related objection. He considers an ensemble of particles in the ground state of a one-dimensional length-*L* “box” potential so that

(47) $$\Psi \left(x_{1},x_{2},\ldots ,x_{N},t\right)=\phi \left(x_{1},t\right)\phi \left(x_{2},t\right)\cdots \phi \left(x_{N},t\right)$$

where $\phi \left(x,t\right)=\sqrt{\frac{2}{L}}\sin\left(\pi x/L\right)e^{-iE_{0}t/\hbar }$. Bricmont explains that

“because of the law of large numbers, the set of typical points [for which the statistical distribution of positions in the ensemble approximately matches ${\left|\phi \right|}^{2}$)] will have a ${\left|\Psi \right|}^{2}$-measure close to 1, for *N* large. So, if one picks a microscopic configuration of the universe *Q* that is typical relative to ${\left|\Psi \right|}^{2}$, it will give rise... to an empirical distribution satisfying Born’s statistical law...

“[Dürr, Goldstein, and Zanghí] claim that quantum equilibrium and therefore Born’s law is actually very natural. However, for that last claim to be right, one needs to argue that the measure with respect to which the configurations under discussion are typical is itself ‘natural’ (every configuration is typical with respect to at least one measure—the delta measure concentrated on itself)”.

[44]

Bricmont goes on to state that, for example, on Bayesian grounds, one might reasonably expect a uniform distribution rather than the ${\left|\phi \right|}^{2}$-distribution which vanishes at the edges of the “box”. The implication is that one has not really *explained* the ${\left|\phi \right|}^{2}$ distribution, but only showed that it follows from a typicality measure that has been specifically selected to produce this very result:

“In fact the only ‘explanation’ of the fact that we obtain a ${\left|\phi \right|}^{2}$ distribution rather than a uniform distribution is probably that God likes quantum equilibrium and Born’s law and so put it there at the beginning of times.

“The upshot of this discussion is that quantum equilibrium, in Bohmian mechanics, should, in my view, be presented as a postulate, independent of the other ones, rather than as somehow being the only natural or reasonable choice. It is not a particularly unnatural choice and it is true that quantum equilibrium is still far less mysterious than classical non equilibrium at the origin of the universe... But one should not present it as more natural than it is”.

[44]

The objections here share the following common structure: when the universal wave function has the structure of Equation (37), where there is an ensemble of *n* sub-systems with identical effective wave functions ψ, it is true that μ-most configurations will display empirical statistics consistent with the Born rule, $p={\left|\psi \right|}^{2}$, if the typicality measure is given by $\mu ={\left|\Psi \right|}^{2}$. But it is also true, for example, that μ-most configurations will display (non-Born rule!) $p={\left|\psi \right|}^{4}$ statistics, if, instead, the typicality measure is given by $\mu ={\left|\Psi \right|}^{4}$... and that μ-most configurations will display (differently non-Born rule!) p= constant statistics if, instead, the typicality measure is given by μ= constant... and so on.

The basis for the feeling of circularity is clear: it seems that one simply gets, as the “typical” statistics for ensembles, whatever one wants, and in particular the sub-system equivalent of whatever one uses for the fundamental typicality measure μ: $\mu ={\left|\Psi \right|}^{2}$ gives $p={\left|\psi \right|}^{2}$, but $\mu ={\left|\Psi \right|}^{4}$ gives $p={\left|\psi \right|}^{4}$, etc. Of course, the equivariance of the specific measure $\mu ={\left|\Psi \right|}^{2}$ may suggest that this particular measure is dynamically privileged and hence in some sense “natural”. But, like other “naturalness” arguments in physics, this may seem rather subjective and insufficiently substantial as a foundation for a genuine *explanation* of Born rule statistics.

## 7. Discussion

The debate, between those who think that Born-rule statistics in the pilot-wave theory should be explained by some kind of dynamical relaxation and those who think the Born-rule should be derived by a typicality analysis, has raged for several decades in a rather sectarian way. My view is that both perspectives contain valuable insights and both sides offer critiques of their opponents’ arguments which can sharpen our understanding of the truth. We should look to combine the insights emerging from both sides of the debate, and thereby aim to construct a single unified explanation of the Born-rule, instead of feeling constrained to make a binary, either-or choice between them.

For example, I think it must be recognized that the dynamical relaxation perspective at least tacitly relies on appeals to typicality. It is emphatically *not* the case that *every* initial configuration, consistent with some particular out-of-equilibrium, non-Born-rule distribution, will evolve monotonically toward coarse-grained equilibrium. See, e.g., Figure 5, which shows an ensemble that is initially in coarse-grained equilibrium evolving through a sequence of increasingly *out-of* (-coarse-grained)-equilibrium distributions. In addition, as discussed in Section 4, there is not much comfort in the fact that dynamical relaxation has only been shown to occur for initial distributions respecting the “no fine-grained micro-structure condition” which, strictly speaking, the probability distributions for which the ensembles pictured in frames (a)–(e) of Figure 5 are typical exemplars violate. There *is* no actually-existing universal “probability distribution” P(q). All that exists (in addition to the wave function) is the actual configuration *Q* and its subsets, including *finite* ensembles of similarly-prepared subsystems whose empirical distributions, given by Equation (38), necessarily contain fine-grained micro-structure.

Moreover, such finite-ensemble empirical distributions simply do not allow any meaningful statements about a hypothetical associated continuous probability distribution p(x). For example, what does it even mean to say that the probability distribution p(x)—associated with the ensemble depicted in frame (f) of Figure 2—has *no* fine-grained micro-structure, whereas the p(x) associated with the ensemble depicted in frame (a) of Figure 5 *does* have fine-grained micro-structure? I think such a claim is utterly empty in the final analysis. All one can say is that the two ensembles share the same equilibrium, Born-rule coarse-grained distribution $\bar{p}\left(x\right)={\left|\psi \left(x\right)\right|}^{2}$, but one of them has the property that it *stays* Born-rule distributed (in the coarse-grained sense) whereas the other one (at least for a while!) does not.

As discussed in Section 4, I think these considerations imply that, at most, the claim defended by the dynamical relaxation program must be that relaxation toward coarse-grained equilibrium (i.e., toward FAPP Born-rule statistics) is *typical*: not all configurations (that do not already exhibit Born-rule statistics) will evolve into configurations that do exhibit Born-rule statistics, but the overwhelming majority of them (in some appropriate sense) will.

The sorts of numerical simulations pioneered by Valentini and Westman (and which I have reproduced here for illustrative purposes) strongly suggest that this modified dynamical relaxation claim is *true*: evolution toward coarse-grained sub-quantum equilibrium really is typical, such that, if one believes that the universe may have started in a (globally atypical) configuration for which Born-rule statistics did not obtain, we should expect Born-rule statistics to emerge over time and hence be observed today (As a concrete example in support of this claim, I ran the simulation depicted in Figure 2, several times—with different initial positions randomly drawn from the distribution p= constant—and it looks qualitatively the same every time). But the explicit recognition of the role of typicality in that explanation should also remove most of the motivation for thinking the universe may have started in some “out-of-equilibrium” initial state in the first place, such states representing, after all, a vanishingly small fraction (...at least, if one uses the supposedly “natural” measure of typicality, $\mu ={\left|\Psi \right|}^{2}$...) of those which could evolve into configurations consistent with what is observed today.

Note here the dramatic contrast with the case of thermodynamic equilibrium, for which there is compelling observational evidence that the out-of-thermodynamic-equilibrium state we see today did arise from an earlier state that was even further out-of-equilibrium. In the sub-quantum case that we have been discussing in this paper, all observational evidence to date is consistent with the (sub-) Quantum Equilibrium Hypothesis, and—contrary to claims made sometimes by Valentini (for example, Valentini and Westman write in [34]: “a relaxation process from an earlier non-equilibrium state.... leads naturally to the suggestion that quantum non-equilibrium may have existed in the early Universe...”)—there is no compelling reason to think that today’s equilibrium arose from an earlier state of disequilibrium. The idea that *most* non-equilibrium configurations evolve toward coarse-grained equilibrium (which is how we are suggesting Valentini’s claims should be understood, and which idea we are willing to grant is probably true) does *not* imply that equilibrium is most likely to have arisen from earlier disequilibrium. Just the opposite is true! Concretely, if one randomly chooses a large number of equilibrium ensembles that look like frame (f) of Figure 2 and runs them all backwards in time, very few will look like frame (a) of Figure 2 (or any other disequilibrium distribution) at t=0; the overwhelming majority will still look just like frame (f), i.e., will exhibit equilibrium, Born-rule statistics at the initial time as well.

In any case, the melding of the typicality and dynamical relaxation perspectives seems to cast a calming and clarifying light on the subject. Invoking typicality helps one understand exactly what the dynamical relaxation argument purports to prove, and helps one more fairly assess how promising it might be, for example, to search for violations of Born-rule statistics in relic particles from the early universe.

In addition, conversely, the time-evolution that is the focus of the dynamical relaxation approach—and which is so vividly portrayed in the numerical simulations of [34,35]—also helps clarify the typicality argument in the face of the sorts of objections reviewed in Section 6. It is definitely true that, as long as one simply considers a specific moment in time, the explanation of $p={\left|\psi \right|}^{2}$ by appeal to $\mu ={\left|\Psi \right|}^{2}$ has an unconvincing and circular character. But that single-moment-in-time snapshot is really a thin caricature of the actual argument.

The real argument is that Born rule $p\left(x,t\right)={\left|\psi \left(x,t\right)\right|}^{2}$ statistics—across a range of nonzero times *t*—arise for typical initial configurations X(0) if we measure typicality using $\mu ={\left|\Psi \left(q,0\right)\right|}^{2}$. Note in particular here that it is *not* the case that if one instead measured typicality using, say, the μ= constant measure, one would get p(x,t)= constant! That is, once we move beyond the single-moment-in-time caricature, it is simply not the case that the typical p(x) is the same function of ψ that the typicality measure μ is as a function of Ψ.

Dürr, Goldstein, and Zanghí explain, for example, that one could infer the typicality of the non-Born-rule statistics $p\left(x,t\right)={\left|\psi \left(x,t\right)\right|}^{4}$

“*provided* the sense [μ] of typicality were given, not by ${\left|\Psi \right|}^{4}$ (which is not equivariant), but by the density to which $|\Psi_{t}{|}^{4}$ would backwards evolve as the time decreases from *t* to THE INITIAL TIME 0. This distribution, this sense of typicality, would presumably be extravagantly complicated and exceedingly artificial.

“More important, it would depend upon the time *t* under consideration, while equivariance provides a notion of typicality that works for all *t*”.

[38]

This is an extremely important point. With (non-trivial) time-evolution between the initial time and the time at which one is interested in considering the statistical distribution, one does not simply get out for p(x) what one puts in for μ. Note as well that what DGZ describe here as an “extravagantly complicated and exceedingly artificial” typicality measure μ could also perhaps be described by saying that μ has an implausibly fine-grained micro-structure.

I also find it illuminating to turn Dürr, Goldstein, and Zanghí’s point around and consider the sorts of statistics one would diagnose as typical if one began with a more plausibly smooth (but non-${\left|\Psi \right|}^{2}$) typicality measure, such as μ= constant or $\mu ={\left|\Psi \right|}^{4}$, over initial configurations. Assuming the wave function of the universe has a non-trivial dynamics, such a μ would forward-evolve into something “extravagantly complicated and exceedingly artificial” at later times *t*. Indeed, this is precisely the sort of evolution we have illustrated in, for example, Figure 4: even for an extremely simple (two-dimensional!) system, an initially uniform distribution evolves into something with an incredible degree of fine-grained micro-structure in a very short period of time. This is what Valentini’s relaxation argument predicts, and one thus expects that something qualitatively similar will happen with the (evidently much more complicated!) evolution of the wave function of the entire universe, such that (say) an initially-uniform μ will forward-evolve into something with an incredibly filimented, fine-grained, non-uniform structure which will diagnose, as typical for ensembles of similarly-prepared sub-systems, a p(x,t) function with its own extravagantly complicated fine-grained structure... but whose coarse-grained partner $\bar{p}\left(x,t\right)$ matches the Born rule distribution ${\left|\psi \left(x,t\right)\right|}^{2}$.

Dürr, Goldstein, and Zanghí have shown that Born-rule statistics are to be expected from the pilot-wave theory, if one assesses typicality using the “natural” equivariant measure, $\mu ={\left|\Psi \right|}^{2}$. What I mean to be recommending here is that Valentini’s analysis, and the associated numerical simulations, can be taken as suggesting that other, non-equivariant typicality measures (which are, at t=0, in some sense “reasonably smooth”, i.e., which contain no fine-grained micro-structure, i.e., which are not extravagantly complicated and exceedingly artificial) will *also* end up diagnosing, as typical, Born-rule statistics at later times. So to whatever extent one finds the typicality argument presented by DGZ to be circular, or to rely too heavily on unconvincing “naturalness” type assumptions, one can rest assured that Born-rule statistics are typical—i.e., that one should expect to see Born-rule statistics in practice—not *only* according to that one special equivariant measure of typicality, but according to virtually *any* “reasonable” measure of typicality.

That, of course, does not mean that Born rule statistics are absolutely guaranteed by the pilot-wave theory’s dynamics. They aren’t. There are possible initial configurations of the universe—indeed, there are an infinite number of them—that will, for example, give rise to perfect $p={\left|\psi \right|}^{4}$ statistics today. But these are like the possible states for a box of gas molecules in which every molecule has exactly the same velocity: the existence of such possible states should not undermine one’s expectation to see a Maxwell velocity distribution. Similarly here: I think what the typicality analysis together with Valentini’s relaxation argument and the associated numerical solutions show is that any “reasonable”—smooth, simply-expressable, non-artificial—measure over initial configurations will imply that *we should expect to see Born-rule statistics*. If that does not constitute a genuine statistical *explanation* of the Born rule, from the dynamical first principles of the pilot-wave theory, I truly do not know what would or could.
